# Health Risk of Polonium 210 Ingestion via Drinking Water: An Experience of Malaysia

**DOI:** 10.3390/ijerph15102056

**Published:** 2018-09-20

**Authors:** Minhaz Farid Ahmed, Lubna Alam, Che Abd Rahim Mohamed, Mazlin Bin Mokhtar, Goh Choo Ta

**Affiliations:** 1 Institute for Environment and Development (LESTARI), Universiti Kebangsaan Malaysia (UKM), UKM Bangi 43600, Selangor, Malaysia; minhazhmd@yahoo.com (M.F.A.); mazlin@ukm.edu.my (M.B.M.); gohchoota@ukm.edu.my (G.C.T.); 2 School of Environmental and Natural Resource Sciences, Universiti Kebangsaan Malaysia (UKM), UKM Bangi 43600, Selangor, Malaysia; carmohd@ukm.edu.my

**Keywords:** drinking water, radioactivity, annual effective dose, carcinogenic

## Abstract

The presence of toxic polonium-210 (Po-210) in the environment is due to the decay of primordial uranium-238. Meanwhile, several studies have reported elevated Po-210 radioactivity in the rivers around the world due to both natural and anthropogenic factors. However, the primary source of Po-210 in Langat River, Malaysia might be the natural weathering of granite rock along with mining, agriculture and industrial activities. Hence, this is the first study to determine the Po-210 activity in the drinking water supply chain in the Langat River Basin to simultaneously predict the human health risks of Po-210 ingestion. Therefore, water samples were collected in 2015–2016 from the four stages of the water supply chain to analyze by Alpha Spectrometry. Determined Po-210 activity, along with the influence of environmental parameters such as time-series rainfall, flood incidents and water flow data (2005–2015), was well within the maximum limit for drinking water quality standard proposed by the Ministry of Health Malaysia and World Health Organization. Moreover, the annual effective dose of Po-210 ingestion via drinking water supply chain indicates an acceptable carcinogenic risk for the populations in the Langat Basin at 95% confidence level; however, the estimated annual effective dose at the basin is higher than in many countries. Although several studies assume the carcinogenic risk of Po-210 ingestion to humans for a long time even at low activity, however, there is no significant causal study which links Po-210 ingestion via drinking water and cancer risk of the human. Since the conventional coagulation method is unable to remove Po-210 entirely from the treated water, introducing a two-layer water filtration system at the basin can be useful to achieve SDG target 6.1 of achieving safe drinking water supplies well before 2030, which might also be significant for other countries.

## 1. Introduction

Po-210, a decay element of U-238, is a naturally occurring radionuclide mostly found in water, soil and food. The significant sources of terrestrial radiation are primordial radionuclides, namely U-238, Th-232, and K-40, which are dispersed around the Earth’s crust and widely reported in Peninsular Malaysia [1]. In the Langat River, Po-210 is mainly due the natural weathering of the minerals, uraninite UO_2_ (pitchblend), carnotite, and autunite of the acid intrusive granitic rock [2,3,4]. Therefore, human beings are at risk of ingestion of Po-210 through drinking water and exposure to annual effective doses for a long time. Moreover, Po-210 is considered as one of the primary sources of alpha exposure [5] to human beings through ingestion via drinking water [6,7,8]. Alam and Mohamed [5] also reported Po-210 is about 250,000 times more toxic than hydrogen cyanide; therefore, it could be carcinogenic if ingested through drinking water [9,10,11,12,13].

Natural sources contribute about 80% to the radionuclides generated in Malaysia, the other sources being anthropogenic ones such as industrial activities as well as mining of natural resources along with gas and petroleum exploration [14]. Many studies in Peninsular Malaysia have investigated the existence of radioactivity from Ra-226, Ra-228, Pb-210, Cs-137, Th-232, K-40 and Rn-222 in water bodies [15,16,17,18] and it was reported that about 80 to 87% of the human exposure to radioactivity in Peninsular Malaysia results mainly from natural sources such as primordial radionuclides, cosmogenic radionuclides, etc. [14,16]. The atmospheric source is also considered as one of the sources of Po-210 in water because of the decay of Rn-222 gas and the decay of naturally occurring U-238 [15,19].

Malaysia has seriously focused on industrial expansion to achieve developed country status by 2020 [20,21]. As a result, the rivers in Malaysia have been found to be severely contaminated with heavy metals [22] as well as radioactive elements [17,23,24]. Rapid mineralization of tin ore from the Main Range Granite is the prime source of the high radioactivity associated with soil in the western region of Peninsular Malaysia [2]. In Selangor state, the maximum activity of U-238, Ra-226, Th-232, and K-40 recorded in sediments from the tin recycling ponds are higher than the rest of Malaysia and is the world’s highest natural activity [25]. The DOE [26] reported that the industrial sector and quarries are responsible for the source points of pollution in the Langat River, quantified at 8.14% and 0.24%, respectively. Therefore, significant sources of primordial radionuclides in the Langat River Basin are sand and gravel extraction 56.21%, and earth material extraction 28.10%, along with granite quarries 13.73% [27].

In Malaysia, the naturally occurring radionuclides are usually generated from the mining and mineral processing industries. For instance, in Selangor, more than 30 illegal sand mining sites, mainly in the Hulu Selangor, Sepang, and Kuala Langat districts have been identified in the last 20 years, while the government has given permits to only 46 sites for sand mining in private lands [28]. In the Langat, Lui, and Semenyih River Sub-basins, the mining area is about 3.28, 10.55, and 4.63 km^2^, respectively, and the mining area trend is increasing [29]. However, the Malaysian government—based on the Atomic Energy Licensing (Radioactive Waste Management) Regulations 2011—allows industries to run if the generation of natural radionuclides remains below 1 Bq/g [30,31]. Similarly, the Ministry of Health Malaysia recommends that the gross alpha (α) and gross beta (β) levels of both in the river and drinking water should not exceed 0.1 Bq/L and 1.0 Bq/L, respectively, as set by the National Standard for Drinking Water Quality [32].

Langat River in Selangor state is not only one of the primary local sources of drinking water [33,34], but rivers are in general the primary sources of drinking water in all Malaysia. Studies on natural radionuclides in the Langat River have reported that the radioactivity of some radionuclides in the river such as U-238 (3.85 Bq/L), Th-232 (1.14 Bq/L), K-40 (145.67 Bq/L) [23], and Ra-226 (0.26 Bq/L) [35] has exceeded the limit of both the gross alpha 0.1 Bq/L and gross beta 1.0 Bq/L emitters set by the Malaysian standard for raw water quality [32]. Similarly, are only a few studies on the radioactivity of radionuclides in the drinking water of Malaysia, which were reported safe through ingestion [36]. However, in some places of Malaysia the activity of Ra-226, i.e., 0.30 Bq/L in drinking water was recorded above the standard set by the United States Environmental Protection Agency. Moreover, the range of annual effective doses from radionuclides in some places of Malaysia was determined to be 0.02 mSv/year to 0.06 mSv/year [19,37].

As there is no significant study on Po-210 in the drinking water supply chain of Malaysia, there is an urgency to study the Po-210 activity in drinking water because of its high radiotoxicity. Since the Langat River is situated on the acid intrusive granite rock belt that is the prime source of U-238 including Po-210, determining the Po-210 activity in the drinking water and its annual effective dose to humans through ingestion is very important concerning human health carcinogenicity risks. Therefore, this study investigated the Po-210 activity in the drinking water of Langat River Basin and the annual effective dose through ingestion via drinking water.

## 2. Materials and Methods

### 2.1. Water Sample Collection

**Figure 1:**
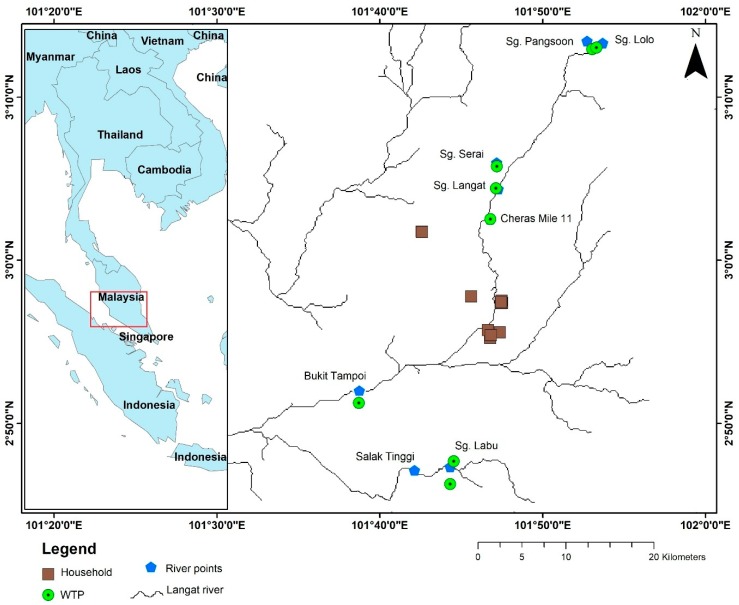
Water sampling points at Langat River Basin, Malaysia.

There are a total nine water treatment plants (WTPs) in the Langat River Basin that provide drinking water to one-third of the population in Selangor State of Malaysia [33] through the water distributor Syarikat Bekalan Air Selangor Sdn Bhd (SYABAS). Although each WTP has been designed to supply treated water in the specific areas of the basin, however, during water shortages treated water from one plant may be channeled to the areas of other plants using the same pipeline. Therefore, twenty liter water samples were collected in polyethylene containers from the Langat River precisely from where the WTPs are located, except the Semenyih WTP were we could not collect samples of raw water for treatment purposes in 2015 (Figure 1) during the rainy days. Similarly, twenty liter treated water samples were collected from the outlets of the WTPs on the same day of the raw water collection. Twenty liters of tap water from five households in the basin as well as three liters of water after filtration were also collected from the same households based on five types of household water filtration systems commonly used in the basin during the same period. Moreover, the collected water samples were analyzed before the end of the half-life of Po-210 i.e., 138 days. Overall, no special measures have been taken to analyze the samples.

### 2.2. Analysis of Po-210

**Figure 2:**
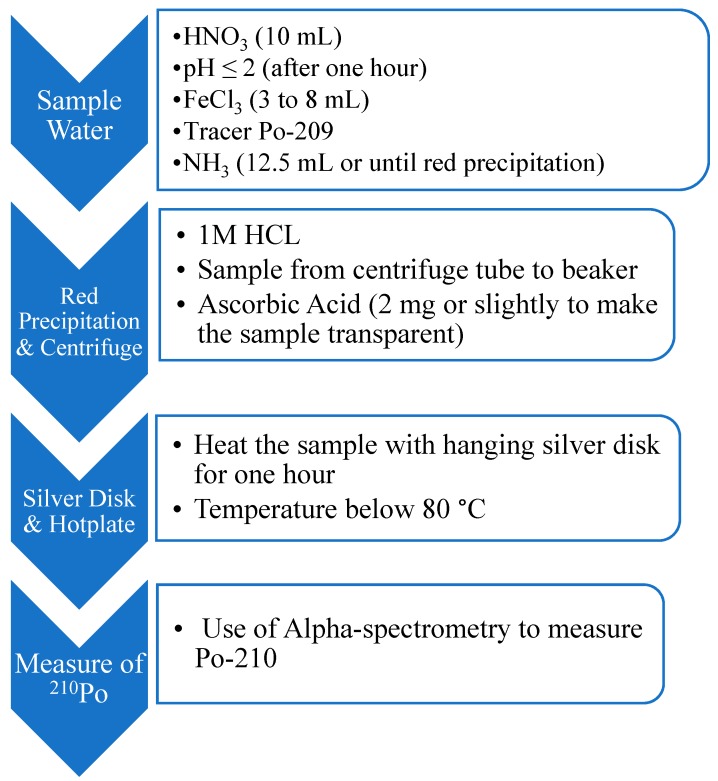
Steps of chemical analysis of Po-210 for fresh water sample [38].

Water samples were filtered with the ADVANTEC membrane filter (pore size: 0.45 μm) and acidified with concentrated HNO_3_ immediately after collection in order to maintain pH ≤ 2 and to avoid contamination. Then the water samples were analyzed for Po-210 following the modified International Atomic Energy Agency (IAEA) method of FeOH_2_ precipitation (Figure 2) [38,39]. Similarly, the activity concentrations of Po-210 in water samples were determined by a CANBERRA Apex Alpha Spectrometer (Canberra Industries, Meriden, CT, USA) with a minimum detectable activity (MDA) of less than 0.2 mBq for a counting efficiency of 12.79%.

A Po-210 tracer has been used in this study to calculate the recovery though using Equation (1) [40].

Po-209 Recovery (%) = [{cpm (tracer)/Counting Efficiency}/Spiked tracer Po-209 (dpm)] × 100(1)

Therefore, the following amounts of tracer Po-209 have been spiked for each sample (Table 1).

### 2.3. Calculating Po-210 Activity

Equation (2) [40] has been used to calculate the activity of Po-210 in the water sampling date (Table 2):

(2) $$\;A_{0}=\frac{A}{e^{-\lambda t}}$$

where $A_{0}$ = Po-210 activity during sampling date, *A* = Po-210 activity during counting date in alpha-spectrometry, *λ* = Coefficient value of Po-210 is 0.005 per year, *t* = Half-life of Po-210 is 138 days

Equation (3) [38] has been used to calculate the Po-210 activity in the counting date of samples by Alpha-spectrometry:

A = {cpm (sample)/cpm (tracer)} × [{Spiked tracer Po-209 (dpm)} × {1/Volume of sample (L)}](3)

where, cpm = Counts per minute; Spiked trace Po-209 (dpm) = {Spiked weight of tracer Po-209 (g) × Stock of trace Po-209 (dpm/g)}; dpm = Disintegrations per minute.

### 2.4. Calculating Annual Effective Dose of Po-210

The annual effective dose of Po-210 to an individual due to ingestion through drinking water was estimated using Equation (4) [24,36]:

D_w_ = C_w_ × CR_w_ × Dc_w_ (4)

where D_w_ = Annual effective dose (mSv/year), C_w_ = Activity of Po-210 in the ingested water (Bq/L), CR_w_ = Annual intake of drinking water (i.e., average 1.996 L/Day by an individual in the Langat Basin through questionnaire survey, so 728.54 L/year) and Dc_w_ = Ingested dose conversion factor for Po-210, which is 1.2 × 10^−3^ mSv/Bq based on the report of International Commission on Radiological Protection (ICRP) 1996 [41,42].

### 2.5. Household Questionnaire Survey

According to the latest population census by the Department of Statistic Malaysia, the total number of households [43] in the Langat River Basin (Malaysia) is 1,494,865. Therefore, 402 household questionnaire surveys were conducted in the basin using Equation (5) [44,45] to get the average daily drinking water intake by the population at the basin to calculate the annual effective dose of Po-210 ingestion through drinking water (Equation (4)).

(5) $$n=\frac{N}{1+N{\left(e\right)}^{2}}$$

where *n* = sample size; *N* = population size; *e* = level of precision, i.e., 0.05 at 95% confidence level.

### 2.6. Time-Series Environmental Data

Flood Incidents data (2005–2016) for the Langat River Basin and water flow data (2005–2015) in the Langat River are obtained from the Dept. of Drainage and Irrigation Malaysia. Similarly, rainfall data (2006–2015) for the basin was collected from the Malaysian Meteorological Department. These data are plotted in the graphs below to show their trends and influences on the Po-210 activity in the Langat River Basin.

## 3. Results and Discussion

### 3.1. Po-210 Status in Raw and Treated Water

Po-210 activity ranges in the river 0.63 ± 0.29 mBq/L to 14.98 ± 1.18 mBq/L and treated water 0.34 ± 0.10 mBq/L to 6.80 ± 0.71 mBq/L (Table 3) of water treatment plants (WTPs) were within the maximum limit of raw and drinking water quality guideline of the Ministry of Health Malaysia [32] and the World Health Organization [41] i.e., 0.1 × 10^3^ mBq/L. Although Po-210 status in raw (*t* = 3.22, *p* = 0.015) and treated water (*t* = 2.924, *p* = 0.022) was significantly safe at 95% confidence level (i.e., *t* statistic) through drinking water, however, the overall efficiency of all the WTPs in the basin was about 59% which might be due to an inability to remove Po-210 from treated water by the conventional method used at the plants as well as the small number of water samples. The Serai and Langat WTPs have the higher Po-210 removal efficiencies from treated water, about 93% and 81%, respectively, might be because of lower iron concentration (175 µg/L) in the upstream region of the Langat River than the downstream (264 µg/L) [46] along with higher manganese and total dissolved solids at the downstream [47,48], although all the WTPs in the basin follow a conventional coagulation method.

#### Sources of Po-210 in the Langat River

**Figure 3:**
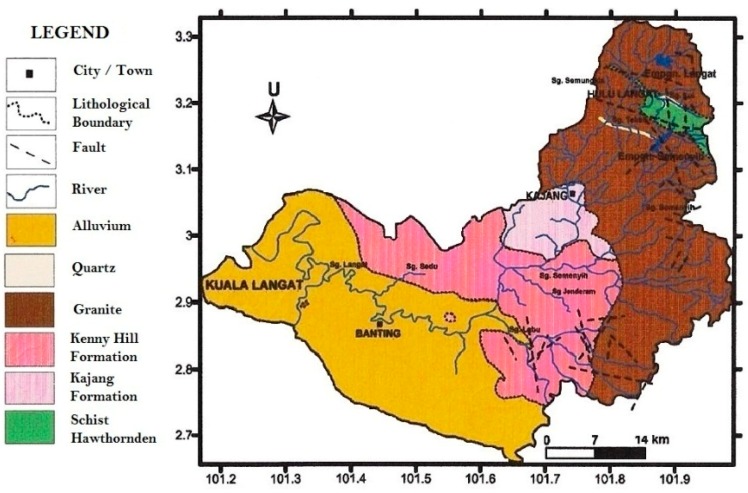
Geological map of Langat River Basin, Malaysia [4].

The Langat River originates at the hilly areas of Hulu Langat, Selangor, Malaysia that are an extension of the Titiwangsa Mountain Range at the north of Selangor. Hence the river drains towards west via the highly urbanized areas of Malaysia until it flows into the Strait of Malacca. Granite rock, extended from the granite batholith of Peninsular Thailand, is widespread underneath central Peninsular Malaysia, especially the entire Selangor state that is situated on two types of lithology based on intrusive rock, i.e., the formation is mainly from gabbro >500 million years ago [2,3]. Therefore, the mean dose rate of soil in Selangor is 183 ± 84 nGy/h with the range of 17.4 nGy/h to 500 nGy/h and the mean value is two times higher than the rest of Malaysia as well as the world average (i.e., 92 nGy/h) [3]. Moreover, a higher dose rate of 500 nGy/h has been recorded in Hulu Gombak because of its location in the acid intrusive granite rock in the Titiwangsa Mountain Range of East Selangor (Figure 3). Hence, through several steps such as weathering, erosion, etc. of granite, quartz rocks [4], these natural radionuclides usually migrate into the Langat River and increase the activity of radionuclides.

However, the higher Po-210 activity in the midstream of Langat River than the upstream might be due to the aerial inputs of the radionuclide, accumulation of radionuclide-rich silt and organic matter, and increased biological production [50] as well as agricultural, industrial and urban waste discharge in the river [51]; since dissolved oxygen (DO) along with a declining trend (Table 4) and conductivity shows a strongly significant inverse correlation (*r* = −0.811) at 99% confidence level (Table 5).

**Figure 4:**
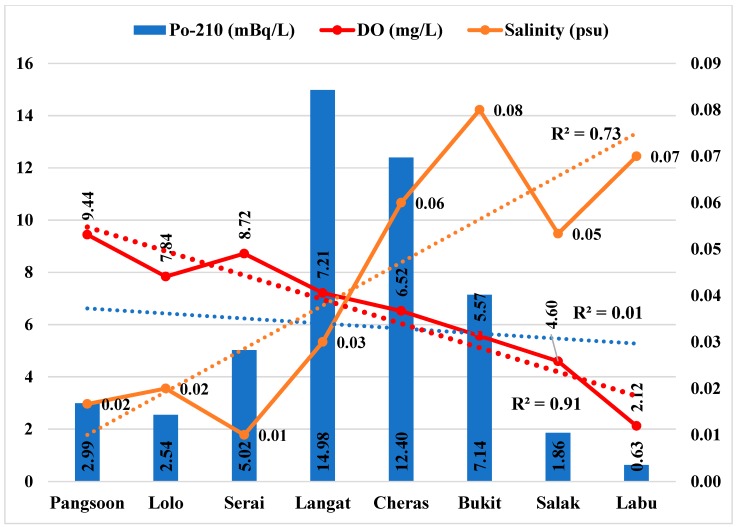
Po-210 activity in Langat River in relation with environmental parameters. Note: R^2^ indicates the variance that is explained by the best-fit line from the upstream to the downstream of the Langat River.

Moreover, human activities such as mining, aquaculture, agriculture, and industrial effluent discharged into the water also contribute to enhancing the levels of radionuclides in the water body [52]. Contrary, the inadequate collaboration among agencies has made the pollution management of river very complex since the river drains through three different constituencies [53,54]. Therefore, the lower Po-210 activity in the raw water at the upstream of Langat River might be due to higher DO as well as non-conservative characteristics of Po-210 [38,55]. Therefore, both the DO (*b* = −0.95, *p* < 0.01) and Po-210 (*b* = −0.09, *p* > 0.10) show declining trends based on the linear regression (Figure 4, Table 4) from upstream to downstream at the Langat River. However, salinity (*b* = 0.85, *p* < 0.01) shows an increasing trend from upstream to downstream at the Langat River. It indicates that the Po-210 might have precipitated due to the increasing salinity towards downstream while mixing with the sea water.

**Figure 5:**
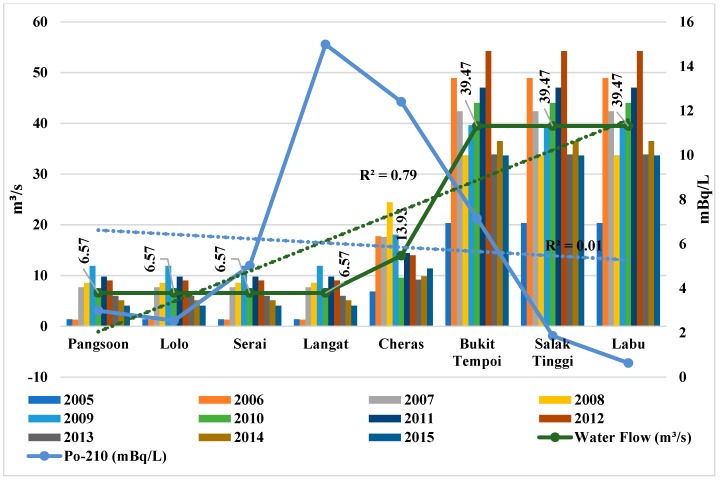
Po-210 activity in Langat River in relation with water flow in the Langat River. Note: Average water flow data (2005–2015) are from the Dept. of Drainage and Irrigation Malaysia. R^2^ indicates the variance that is explained by the best-fit line from the upstream to the downstream of the Langat River.

**Figure 6:**
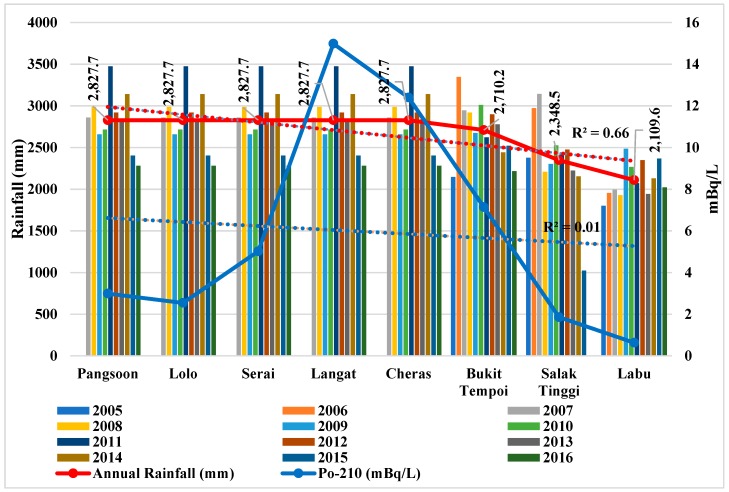
Po-210 activity in Langat River in relation with rainfall in the Langat Basin. Note: Mean rainfall data (2006–2015) from the Malaysian Meteorological Department. R^2^ indicates the variance that is explained by the best-fit line from the upstream to the downstream of the Langat River.

**Figure 7:**
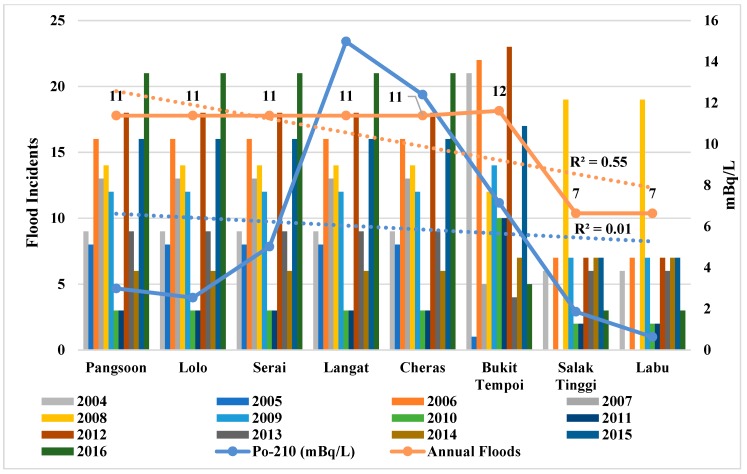
Po-210 activity in Langat River in relation with flood incidents in the Langat Basin. Note: Average flood Incidents data (2005–2016) from the Dept. of Drainage and Irrigation Malaysia. R^2^ indicates the variance that is explained by the best-fit line from the upstream to the downstream of the Langat River.

Similarly, the increasing water flow (*b* = 0.89, *p* < 0.01) at the downstream (Figure 5, Table 4) might have also diluted Po-210 activity in dissolved phase. Moreover, Po-210 activity and water flow in the Langat River show a negative correlation (*r* = −0.370; Table 3). However, the declining rainfall trend (*b* = −0.81, *p* < 0.01) (Figure 6, Table 4) and flood incidents (*b* = −0.74, *p* < 0.05) (Figure 7, Table 4) trend towards downstream of Langat River indicates less atmospheric and terrestrial inputs to have lower Po-210 activity towards downstream than the upstream to midstream. Therefore, Po-210 activity shows a positive correlation (Table 5) with rainfall (*r* = 0.553; *p* = 0.077) and flood incidents (*r* = 0.542; *p* = 0.083) that means there will be lower Po-210 activity in Langat River if there are less rainfall and flood incidents.

Table 3 describes the influence of environmental and physiochemical parameters on the Po-210 activity in the Langat River. Significant positive correlations between Po-210 activity and rainfall (*r* = 0.553 *) as well as Po-210 activity and flood incidents (*r* = 0.542 *) are observed at the 0.10 level, respectively, in the Langat River, that might be because of their decreasing trends towards downstream. The decreasing trend of rainfall during 2005 to 2016 (Figure 6) and flood incidents during 2004 to 2016 (Figure 7) in the Langat River from upstream to downstream also supports the positive correlations between Po-210 and rainfall as well as Po-210 and flood incidents since there are less atmospheric and terrestrial inputs into the river. Accordingly, based on the geochemical behavior of Po-210, it precipitates toward downstream while mixing with the sea water [56]. Therefore, these findings indicate when there is less terrestrial runoff in Langat River, then there might be less attribution from natural and manmade sources to have lower Po-210 activity in raw water.

The activity of Po-210, U-238 decay series progeny, i.e., 14.98 ± 1.18 mBq/L, in the Langat River is higher among many rivers around the world (Table 6); this might be because the river runs through a former tin mining area that can be the source of Naturally Occurring Radioactive Materials (NORM) [57,58]. The other reasons for the enhanced Po-210 activity in the Langat River might be due to the weathering process on the granitic formation of the river basin, the origin of the water, flow rate, flux of radionuclides, and geological characteristics of the area along with haze events in dry seasons [59,60]. The sources of radionuclide pollutants are also from fertilizers, tin mining, aquaculture feed [61] and industries [57]. Similarly, the higher Po-210 activity in the Vistula River, Poland 0.5 ± 0.1 to 9.8 ± 0.02 mBq/L, is mainly because of higher contamination from phosphate fertilizer and coal mining, along with natural sources [62].

However, the world average activity of Po-210 in the drinking water from private wells is 7 to 48 mBq/L [63]. In Malaysia, rivers provide about 98% of the drinking water sources [64,65] and in the case of the Langat River, it provides drinking water to over one-third of the population in the state of Selangor [47,66]. Therefore, the annual effective doses through ingestion of Po-210 by drinking water could be dangerous for human health.

Moreover, among the rivers of Malaysia, the annual effective dose of Po-210 from the Langat River is estimated the highest, i.e., 1.31 × 10^−2^ mSv/year (Table 7), because the river is located in a high rich uranium granite rock belt, although it is below the standard of 0.01 mSv/year. Comparison of the annual effective dose of Po-210 from the Langat River with other rivers around the world indicates that the Langat River is in the second position, while the Vistula River in Poland, i.e., 8.6 × 10^−3^ mSv/year, is in the first position [62].

### 3.2. Po-210 Status in Household’s Tap and Filtration Water

Po-210 activity ranges in tap water 1.05 ± 0.33 mBq/L to 22.35 ± 1.67 mBq/L and filtration water 0.49 ± 0.19 mBq/L to 7.30 ± 0.84 mBq/L at the household level in the Langat Basin were fairly within the maximum limit of drinking water quality standard of MOH and WHO i.e., <0.1 × 10^3^ mBq/L (Table 8). However, the higher mean Po-210 activity in tap water than the raw and treated water indicates contamination in the drinking water distribution pipeline. Terrestrial inputs might have elevated the Po-210 activity in the tap water through the heavy repairing activities of the water distribution pipeline while water was mixing with the soil at Hentian Kajang and UKM areas within the basin. The soil in the granite rock of Peninsular Malaysia including the Langat River Basin highly influences the radioactivity of naturally occurring radionuclides [2,4]. Similarly, the small number of water samples might be one of the reasons of higher Po-210 activity in tap and filtration water as well as the standard deviations. Fortunately, the effectiveness of the household water filtration systems to remove Po-210 from the drinking water in the basin is about 74% (Table 6). Among the commonly used household water filtration systems in the basin, the ultraviolet (UV) filtration system shows higher efficiency (93%) followed by distilled filtration system (69%) in removing Po-210 from drinking water and the Po-210 activity in drinking water at Langat River Basin is within both the national and international drinking water quality guidelines.

Ahmed et al. [67] reported that the Po-210 activity in the supplied water (1.7 mBq/L) in the Langat River Basin was slightly higher than the activity of the treated water 1.5 mBq/L at the outlet of water treatment plants might be due to the contamination in the pipelines of the drinking water supply system. Moreover, the activity of Po-210 in the supplied water in Malaysia is higher than many countries of the world such as 1.0 mBq/L in Italy [70], 2 to 15.2 mBq/L in Hungary [6], 0.48 mBq/L in Poland [71], 1.4 mBq/L in India [72], etc. (Table 9). On the other hand, Po-210 activity in the supplied water of USA 5 mBq/L [73], Italy 3.25 mBq/L [9] and Bombay, India 1.9 mBq/L [72] were higher than the Po-210 activity in the supplied water of Malaysia, 2015 (Table 7).

### 3.3. Human Health Hazard of Po-210 Ingestion

The annual effective doses of Po-210 through ingestion via drinking water are significant in terms of dose contributions. Although the radioactivity of Po-210 in drinking water is lower than the activity of the isotopes of uranium, however Po-210 is considered highly toxic. Moreover, Po-210 is the most important dose contributor through ingestion of drinking water. For instance, Jia et al. [9] investigated that the mean Po-210 activity 3.25 mBq/L and the Po-210 activity in drinking water remained in the last position in the series of U-238, Ra-228 and Pb-210, however in terms of annual effective dose 2.84 × 10^−3^ mSv/year Po-210 was the highest contributor in the same series of radionuclides (Table 9). Unfortunately, the Po-210 activity in the drinking water and its annual effective dose exposure for a long time to human beings has not been studied extensively due the difficulties in determining the radioactivity [19].

Therefore, the calculated annual effective dose of Po-210 ingestion via tap water 7.41 × 10^−3^ mSv/year and household filtration water 1.90 × 10^−3^ mSv/year indicate an acceptable carcinogenic risk for the populations at the Langat River Basin, since the values are within the standard of UNSCAER < 0.12 mSv/year, WHO < 0.01 mSv/year, and ICRP < 1.0 mSv/year [36] and the Polish Ministry of Health < 0.01 mSv/year [75,76] (Table 10). Although the higher standard deviations might be due to the small number of environmental samples, however, the result indicates safe for human consumption. Similarly, the annual effective dose of river 5.20 × 10^−3^ ± 4.56 × 10^−3^ mSv/year and treated water 2.14 × 10^−3^ ± 2.07 × 10^−3^ mSv/year were significantly within the standard limit at 95% confidence level.

A few studies have predicted chronic human health risk of Po-210 ingestion via drinking water [9,10,42,71,77]. Although Zaga et al. [77] estimated Po-210 inhalation and lung cancer, however, there is no significant association study between ingestion of Po-210 via drinking water and types of cancer in human. Luckily, Scott [78] has estimated acute risk of Po-210 ingestion through experimenting on the animal. Scott [78] concluded that: (1) ingestion (or inhalation) of a few tenths of a milligram of Po-210 will likely be fatal to all exposed persons; (2) Lethal intakes are expected to involve fatal damage to the bone marrow which is likely to be compounded by damage caused by higher doses to other organs including the kidneys and liver; (3) Lethal intakes are expected to cause severe damage to the kidney, spleen, stomach, small and large intestines, lymph nodes, skin, and testes (males) in addition to the fatal damage to bone marrow; (4) The time distribution of deaths is expected to depend on the level of radioactivity ingested or inhaled.

Therefore, natural radioactivity ingestion is one of the main causes of human radiation exposure (i.e., global average is 2.4 mSv/year) and it should not exceed 0.1 mSv/year for drinking water according to the European Union Drinking Water Directives (DWD) 98/83/EC [79]. The naturally existing radionuclides in the U-238, Th-232 series, and K-40 are present everywhere in the Earth’s crust. Ra-226, U-238 decay series progeny, and K-40 receive more attention due to their high solubility and mobility [80]. In fact, the radioactivity of radionuclides such as U-238, Th-232, and K-40 is very common in water, soil, and rocks [17]. Hence, it presents a serious threat of radiation to the population if ingested [23]. However, the amount of radioactivity of radionuclides in the surface water varies among different places in the world because of territorial and atmospheric sources [81] as well as man-made activities [16]. The proportion of natural and artificial radioactivity rates on earth is 85%:15% [69], whereas in Malaysia the proportion is 80%:20% [16].

## 4. Conclusions

Po-210 activity in the water supply chain of Langat Basin, Malaysia was within the drinking water quality standard of the Ministry of Health Malaysia and World Health Organization. However, Po-210 activity in the range of 0.63 ± 0.29 to 14.98 ± 1.18 mBq/L in the Langat River is higher than many countries around the world. The Langat River drains through the granite zone, an extension of the granite batholith series of Peninsular Thailand underneath the basin. Therefore, terrestrial sources are the main contributors of primordial radionuclides, such as U-238 and its progeny Po-210 along with the atmospheric and anthropogenic sources to elevate Po-210 activity in the Langat River. Moreover, the less efficiency i.e., an average 59% of conventional water treatment method to remove Po-210 entirely from the treated water might be the reason of higher annual effective dose of Po-210 ingestion via drinking water i.e., via tap water 7.41 ×10^−3^ mSv/year than many countries around the world, even though the limit is safe and the carcinogenic risk is acceptable for human beings. Previous studies have already reported Po-210 is 250,000 times more toxic than hydrogen cyanide, hence it can be carcinogenic to humans if ingested via drinking water for a long time. Moreover, the treated water might become contaminated in the long pipelines in between treatment plants and households. Therefore, a two-layer water filtration system (i.e., effective filtration system at the plants as well as at the household level) should be introduced in the basin to obtain the status of a developed nation by 2020 as well as to achieve the SDG target 6.1 of getting safe drinking water supply well before 2030.

## Figures and Tables

**Table 1:** Amount of Spiked Po-209 Tracer.

Sample	Spiked Weight of Po-209 (g)	Stock Tracer Po-209 (dpm/g)	Spiked Tracer Po-209 (dpm)
Pangsoon River	0.2893	12.17	3.52
Pangsoon WTP	0.4296	1.43	0.61
Lolo River	0.2957	12.17	3.60
Lolo WTP	0.3036	12.17	3.69
Serai River	0.4987	12.17	6.07
Serai WTP	0.3506	1.43	0.50
Langat River	0.2643	12.17	3.22
Langat WTP	0.4629	12.17	5.63
Cheras River	0.4695	12.17	5.71
Cheras WTP	0.4711	12.17	5.73
Bukit River	0.3597	12.17	4.38
Bukit WTP	0.4844	12.17	5.90
Salak River	0.2297	12.17	2.80
Salak WTP	0.4430	12.17	5.39
Labu River	0.2030	1.43	0.29
Labu WTP	0.4896	12.17	5.96
Carbon Supply Hentian Kajang	0.2768	12.17	3.37
Carbon Filter Hentian Kajang	0.2186	12.17	2.66
Distilled Supply UKM	0.2090	1.43	0.30
Distilled Filter UKM	0.1940	12.17	2.36
RO Supply Hentian Kajang	0.3646	12.00	4.38
RO Filter Hentian Kajang	0.2097	12.00	2.52
Alkaline Supply Serdand	0.3289	12.17	4.00
Alkaline Filter Serdand	0.3251	12.17	3.96
UV Supply UKM	0.1880	12.17	2.29
UV Filter UKM	0.3090	12.17	3.76

**Table 2:** Tracer Po-209 recovery (%) in drinking water supply chain samples in the Langat Basin.

Location	Water (L)	Recovery Po-209 (%)	Sampling Date	Test Date
Pangsoon River	20	19.68	6/8/2015	22/12/2015
Pangsoon WTP	20	26.43	6/8/2015	22/12/2015
Lolo River	20	19.27	6/8/2015	22/12/2015
Lolo WTP	20	33.36	6/8/2015	22/12/2015
Serai River	20	15.92	20/8/2015	2/1/2016
Serai WTP	20	58.84	20/8/2015	28/12/2015
Langat River	20	25.96	11/8/2015	22/12/2015
Langat WTP	20	20.13	11/8/2015	22/12/2015
Cheras River	20	28.70	12/8/2015	26/12/2015
Cheras WTP	20	12.87	12/8/2015	24/12/2015
Bukit River	20	16.84	13/8/2015	26/12/2015
Bukit WTP	20	25.23	13/8/2015	22/12/2015
Salak River	20	14.38	14/8/2015	23/1/2016
Salak WTP	20	11.37	14/8/2015	22/12/2015
Labu River	20	19.70	20/8/2015	2/1/2016
Labu WTP	20	20.62	20/8/2015	2/1/2016
Carbon Supply Hentian Kajang	3	44.09	20/11/2015	28/12/2015
Carbon Filter Hentian Kajang	3	18.81	20/11/2015	28/12/2015
Distilled Supply UKM	3	30.22	20/11/2015	26/12/2015
Distilled Filter UKM	3	23.83	5/5/2016	22/6/2016
RO Supply Hentian Kajang	3	40.20	18/11/2015	24/12/2015
RO Filter Hentian Kajang	3	31.94	18/11/2015	24/12/2015
Alkaline Supply Serdang	3	23.43	15/11/2015	30/1/2016
Alkaline Filter Serdang	3	26.78	15/11/2015	30/1/2016
UV Supply UKM	3	13.38	15/11/2015	30/1/2016
Carbon Supply Hentin Kajang	3	44.09	20/11/2015	28/12/2015
Carbon Filter Hentian Kajang	3	18.81	20/11/2015	28/12/2015

Note: WTP = Water Treatment Plant.

**Table 3:** Po-210 activity (mBq/L) in raw and treated water at the Langat River Basin.

Location	River	WTP	Efficiency WTP (%)	Weighted ^1^ Efficiency
Pangsoon	2.99 ± 0.33	1.22 ± 0.49	59	3.72
Lolo	2.54 ± 0.31	1.83 ± 0.20	28	1.49
Serai	5.02 ± 0.47	0.34 ± 0.10	93	9.83
Langat	14.98 ± 1.18	2.86 ± 0.31	81	25.49
Cheras	12.40 ± 0.84	6.80 ± 0.71	45	11.77
Bukit	7.14 ± 0.69	5.18 ± 0.42	27	4.12
Salak	1.86 ± 0.45	0.84 ± 0.20	55	2.14
Labu	0.63 ± 0.29	0.51 ± 0.11	20	0.26
Mean	5.95 ± 5.22	2.45 ± 2.37	59	58.83 (Total)
*t* value	3.220	2.924		
*p* value	0.015 *	0.022 *		
MOH (2010)	<0.1 × 10^3^ mBq/L			
WOH (2017)	<0.1 × 10^3^ mBq/L			

Note: Reported Po-210 activity is in the sampling date (A_o_) and the standard deviation calculation are based on the radiochemistry method, not on the replicates [49]. ^1^ Sum of the river Po-210 activity of the eight sampling points has been taken as the basis of weighted average for each WTP. * Significant at 95% confidence level.

**Table 4:** Results of the Linear Regression-based Trend line of Po-210, Physiochemical and Environmental Parameters.

Parameters	Constant	River Point Coefficient	R-Square	F-Stat
Po-210	6.81 (1.56)	−0.09 (−0.22)	0.01	0.05 (0.832)
DO	10.66 (17.43)	−0.95 (−7.63)	0.91	58.21 (0.0003)
Salinity	0.001 (0.61)	0.85 (4.01)	0.73	16.09 (0.007)
Water Flow	−6.99 (−1.09)	0.89 (4.72)	0.79	22.28 (0.003)
Rainfall	3079.94 (22.62)	−0.81 (−3.43)	0.66	11.79 (0.014)
Flood Incidents	13.24 (10.56)	−0.74 (−2.70)	0.55	7.27 (0.036)

**Table 5:** Correlation of water quality and environmental parameters in Langat River.

Parameters		Po-210	Salinity	DO	Conductivity	Temperature	Flood	Rainfall	Water Flow
**Po-210**	Pearson Correlation	1							
	Sig. (1-tailed)								
**Salinity**	Pearson Correlation	0.043	1						
	Sig. (1-tailed)	0.459							
**DO**	Pearson Correlation	0.244	−0.800 ***	1					
	Sig. (1-tailed)	0.280	0.009						
**Conductivity**	Pearson Correlation	0.016	0.995 ***	−0.811 ***	1				
	Sig. (1-tailed)	0.485	0.000	0.007					
**Temperature**	Pearson Correlation	0.161	0.819 ***	−0.852 ***	0.824 ***	1			
	Sig. (1-tailed)	0.352	0.006	0.004	0.006				
**Flood**	Pearson Correlation	0.542 *	−0.287	0.743 *	−0.318	−0.533	1		
	Sig. (1-tailed)	0.083	0.245	0.017	0.222	0.087			
**Rainfall**	Pearson Correlation	0.553 *	−0.546	0.900 ***	−0.556	−0.657 **	0.922 **	1	
	Sig. (1-tailed)	0.077	0.081	0.001	0.076	0.038	0.001		
**Water Flow**	Pearson Correlation	−0.370	0.838 ***	−0.867 ***	0.850 ***	0.815 ***	−0.609	−0.805 ***	1
	Sig. (1-tailed)	0.184	0.005	0.003	0.004	0.007	0.054	0.008	

*** Correlation is significant at the 0.01 level (1-tailed); ** Correlation is significant at the 0.05 level (1-tailed). * Correlation is significant at the 0.10 level (1-tailed).

**Table 6:** Activity of Po-210 (mBq/L) both in Malaysian and various rivers of the world.

River (Year)	Minimum	Maximum	References
Langat, Malaysia	0.63 ± 0.29	14.98 ± 1.18	*Present Study*
Langat, Malaysia (2015)	-	7.70 ± 0.60	[67]
Kuala Selangor, Malaysia (2010)	0.0002 ± 0.0001	0.014 ± 0.003	[38]
Kuala Selangor, Malaysia (2005)	0.22 ± 0.06	0.75 ± 0.28	[68]
Vistula, Poland (2004)	0.49 ± 0.09	9.80 ± 0.02	[62]
Oder, Poland (2004)	0.60 ± 0.09	5.21 ± 0.19	
Pomeranian, Poland (2004)	3.82 ± 0.24	5.50 ± 0.33	
Yellow, China (1999)	0.25 ± 0.08	1.55 ± 0.50	[69]
Tagus, Portugal (1997)	0.50 ± 0.36	0.67 ± 0.03	[70]

**Table 7:** Annual effective dose of Po-210 (mSv/year) based on river water globally.

Location (Year)	Minimum	Maximum	References
Langat, Malaysia	5.51 × 10^−4^	1.31 × 10^−2^	*Present Study*
Langat, Malaysia (2015)	-	6.8 × 10^−3^	[67]
Kuala Selangor, Malaysia (2010)	0.0002 × 10^−3^	0.01 × 10^−3^	[38]
Kuala Selangor, Malaysia (2005)	0.2 × 10^−3^	0.7 × 10^−3^	[68]
Vistula, Poland (2004)	0.4 × 10^−3^	8.6 × 10^−3^	[62]
Oder, Poland (2004)	0.5 × 10^−3^	4.6 × 10^−3^	
Pomeranian, Poland (2004)	3.4 × 10^−3^	4.8 × 10^−3^	
Yellow, China (1999)	0.2 × 10^−3^	1.4 × 10^−3^	[69]
Tagus, Portugal (1997)	0.4 × 10^−3^	0.6 × 10^−3^	[70]

**Table 8:** Po-210 activity (mBq/L) in tap and filter water at Langat River Basin.

Location	Tap Water	Filter Type	Filtration Water	Efficiency Filter (%)	Weighted ^1^ Efficiency
Hentian Kajang	1.33 ± 0.25	Carbon	1.05 ± 0.33	21	0.66
UKM	1.57 ± 0.70	Distilled	0.49 ± 0.19	69	2.55
Hentian Kajang	22.35 ± 1.67	RO	7.30 ± 0.84	67	35.51
Serdang	1.05 ± 0.33	Alkaline	0.87 ± 0.28	17	0.42
UKM	16.08 ± 2.27	UV	1.13 ± 0.37	93	35.28
Mean	8.48 ± 10.10	Mean	2.17 ± 2.88	74	74.42 (Total)
*t* value	1.885	*t* value	1.684		
*p* value	1.32	*p* value	1.68		
MOH (2010)	<0.1 × 10^3^ mBq/L				
WOH (2017)	<0.1 × 10^3^ mBq/L				

Note: Reported Po-210 activity is in the sampling date (A_o_) and Calculation of standard deviation is based on the radiochemistry method, not on the replicates [49]. ^1^ Sum of all the Po-210 activities in all the five-tap water has been taken as the basis of weighted average for each household water filtration system.

**Table 9:** Po-210 activity in household’s tap water around the world.

Location (Year)	Activity (mBq/L)	Dose (mSv/Year)	References
Langat Basin	8.48	7.41 × 10^−3^	*Present Study*
Bangi, Malaysia (2015)	1.7	1.5 × 10^−3^	[67]
Italy (2009)	3.25	2.84 × 10^−3^	[9]
Italy (2007)	1	0.9 × 10^−3^	[70]
Hungary (2010)	2	1.75 × 10^−3^	[6]
Poland (2001)	0.48	0.42 × 10^−3^	[71]
India (2001)	1.4	1.2 × 10^−3^	[72]
Bombay, India (1977)	1.9	1.7 × 10^−3^	
Brazil (1992)	1	0.9 × 10^−3^	
Portugal (1995)	0.21	0.2 × 10^−3^	
Syria (1995)	1	0.9 × 10^−3^	
Austria (2001)	0.4	0.35 × 10^−3^	[74]
USA (2008)	5	4.4 × 10^−3^	[73]

**Table 10:** Annual effective dose of Po-210 via drinking water at Langat River Basin.

Location	River (mSv/Year)	WTP (mSv/Year)	Location	Tap (mSv/Year)	Filter Type	Filtered (mSv/Year)
Pangsoon	2.62 × 10^−3^	1.07 × 10^−3^	Hentian Kajang	1.16 × 10^−3^	Carbon Filter	9.18 × 10^−4^
Lolo	2.22 × 10^−3^	1.6 × 10^−3^	UKM	1.37 × 10^−3^	Distilled Filter	4.28 × 10^−4^
Serai	4.39 × 10^−3^	3.01 × 10^−4^	Hentian Kajang	1.95 × 10^−2^	RO Filter	6.38 × 10^−3^
Langat	1.31 × 10^−2^	2.50 × 10^−3^	Serdang	9.18 × 10^−4^	Alkaline Filter	7.61 × 10^−4^
Cheras	1.08 × 10^−2^	5.94 × 10^−3^	UKM	1.41 × 10^−2^	UV Filter	9.88 × 10^−4^
Bukit	6.24 × 10^−3^	4.53 × 10^−3^	Average	7.41 × 10^−3^	Average	1.90 × 10^−3^
Salak	1.63 × 10^−3^	7.35 × 10^−4^	Std.	8.78 × 10^−3^	Std.	2.52 × 10^−3^
Labu	5.52 × 10^−4^	4.43 × 10^−4^	*t* value	1.886	*t* value	1.684
Average	5.20 × 10^−3^	2.14 × 10^−3^	*p* value	0.132	*p* value	0.167
Std.	4.56 × 10^−3^	2.07 × 10^−3^				
*t* value	3.223	2.926				
*p* value	0.015 *	0.022 *				

Note: * Significant at 95% confidence level. United Nations Scientific Committee on Effects of Atomic Radiation (UNSCAER) < 0.12 mSv/year. World Health Organization (WHO) < 0.01 mSv/year. International Commission on Radiological Protection (ICRP) < 1.0 mSv/year. Polish Ministry of Health < 0.01 mSv/year.
